# Barriers to the use of no and low alcohol products in high‐risk drinkers

**DOI:** 10.1111/dar.14006

**Published:** 2025-02-09

**Authors:** Emma L. Davies, Parvati Perman‐Howe, Jennifer Seddon, Timothy Piatkowski, Cheneal Puljevic, Monica J. Barratt, Adam R. Winstock, Jason A. Ferris

**Affiliations:** ^1^ Centre for Psychological Research Oxford Brookes University Oxford UK; ^2^ School of Medicine and Population Health University of Sheffield Sheffield UK; ^3^ School of Applied Psychology and Griffith Centre for Mental Health Griffith University Brisbane Australia; ^4^ School of Public Health The University of Queensland Brisbane Australia; ^5^ Social Equity Research Centre and Digital Ethnography Research Centre RMIT University Melbourne Australia; ^6^ National Drug and Alcohol Research Centre UNSW Sydney Sydney Australia; ^7^ University College London, UK, Global Drug Survey London UK; ^8^ Centre for Health Services Research The University of Queensland Brisbane Australia

**Keywords:** alcohol free, barriers, COM‐B, high risk drinker, low alcohol

## Abstract

**Introduction:**

Consuming no or low (NoLo) alcohol products in place of regular strength alcohol products could reduce alcohol‐related harms in high risk drinkers. This study provides a new perspective by exploring beliefs about NoLo products and motives for their use by level of risky drinking using a model of behaviour change.

**Methods:**

The 2022 Global Drug Survey included items on NoLo product use, beliefs, and motives for consuming or not consuming NoLo products. Findings were mapped onto the COM‐B (capability‐opportunity‐motivation) model.

**Results:**

In a sample of 33,033 respondents (59.5% cis men; 37.3% cis women; 3.2% trans/non‐binary) over half (52.2%) reported NoLo product use in the last 12 months. Recent NoLo use was associated with older age, employment status and more common in respondents who drank alcohol compared to non‐drinkers. High‐risk drinkers were more likely to believe NoLo products could help them to drink less and to avoid embarrassment. However, higher risk drinkers who had never consumed NoLo products were more likely to report that they drank to be intoxicated and believed they would not have a good time if they switched.

**Discussion and Conclusions:**

People who are drinking for enhancement motives (e.g., for fun, to feel intoxicated) may be less amenable to substituting regular strength alcohol products for NoLo products. NoLo use may help some higher risk drinkers consume less alcohol, and social and motivational factors could be targeted to increase their use. There should be renewed focus on broader intervention strategies, such as creating viable social alternatives to consuming alcohol.

**Key Points**

- This study is a novel application of the COM‐B model to no or low alcohol (NoLo) product use in high‐risk drinkers.
- High‐risk drinkers who consumed NoLo products endorsed health and social motives.
- Barriers to NoLo use included prompting alcohol consumption in high‐risk drinkers.
- High‐risk drinkers sought intoxication and believed NoLos products were less fun.
- Social factors and beliefs could be targeted to increase NoLo substitution in high‐risk drinkers.

## 1 INTRODUCTION

Alcohol use is a leading global risk factor for poor health [1]. In recent years, there has been a focus on the potential benefits of encouraging consumers to switch regular strength alcohol products for alcohol free and low strength (NoLo) alcohol products [2]. NoLo products are defined as containing no more than 1.2% alcohol by volume [3]. NoLo product sales have increased in the last 10 years, although market share remains low [4, 5]. While there is a lack of evidence about their impacts, there are a number of potential benefits and drawbacks to this trend.

Using NoLo products as substitutes for regular strength products may reduce alcohol consumption amongst consumers [6, 7]. They may allow consumers to experience the social benefits of drinking with others [8], without the stigma that non‐drinkers sometimes feel [9, 10]. In a recent UK study, women who had stopped drinking alcohol reported that NoLo products had been helpful as they led to fewer challenges from peers about why they were not drinking alcohol [11]. In a US study, respondents with alcohol use disorder (AUD) were more likely than those without AUD to consume NoLo products to help them to abstain from drinking [12].

Despite the benefits, there are concerns that NoLo products have the potential to reinforce or widen existing health inequalities. Research has shown that NoLo consumers tend to be more affluent [5, 13, 14]. This may in part, be because NoLo products are often more expensive than alcohol products [4].

NoLo products may reinforce the notion that alcohol has a place in almost any occasion, thus, embedding it further into sociocultural norms [15]. Brand sharing between NoLo drinks and regular strength drinks mean that alcohol producers can circumnavigate advertising restrictions [16]. Such ‘surrogate’ marketing [17], where promotion of NoLo drinks closely resemble their regular strength counterparts, may be triggering for people in recovery from AUD and those trying to cut down. It has been suggested that NoLo advertising promotes the notion of NoLo consumption as temporary, and only for specific contexts, not to replace usual drinking practices [18]. Interestingly, therefore, some research has shown that NoLo products are purchased by those that buy the most alcohol [13] and that NoLo consumers tend to be heavier drinkers [12, 14].

Research into the use of NoLo products and the associated public health impacts is still emerging, but, if used as substitutes for regular strength products, they have potential to contribute to harm reduction. It is, therefore, timely to understand what could motivate risky drinkers to substitute regular strength products for NoLo products. The incentive motivation model is widely used to explore motives for drinking [19, 20]. Approach/avoidance goals and internal/external factors are brought together to produce four key motivators: conformity (external, negative); coping (internal, negative); enhancement (internal, positive); and social (external, positive) [21]. Enhancement motives, such as the desire to feel intoxicated may inhibit NoLo use, but those drinking to conform in a social setting may find NoLo products helpful.

To explore possible motives and barriers to NoLo product use, we employed an overarching model of behaviour—the capability, opportunity, motivation, behaviour (COM‐B) model [22]. The COM‐B model proposes that behaviour is the result of a dynamic combination of an individual's capability, opportunity, and motivation. Capability may be physical (e.g., skill, strength) or psychological (e.g., knowledge, psychological stamina). Opportunity may be physical (in terms of the environment, time or resources) or social (norms, cues, interpersonal influences). Motivation may be reflective (plans or conscious intentions) or automatic (reactions, habits, desires and impulses). To explore potential mechanisms of behaviour change we also apply the Theoretical Domains Framework (TDF) [23]. The TDF synthesises key theoretical constructs from other theories of behaviour change, and links them to the COM‐B. Understanding these factors can inform recommendations for interventions and policy development relating to NoLo product use.

We explored three novel research questions (RQ):

Do demographic factors differ across NoLo product use (never/more than 12 months ago/in the last 12 months)?

Do beliefs about NoLo products and reason for their use/non‐use differ by level of risky drinking?

What COM‐B factors could be targeted in order to encourage higher risk drinkers to drink NoLo products in place of regular strength products?

## 2 METHODS

### 2.1 Design

The Global Drug Survey (GDS) is an anonymous, online, cross sectional survey of drug use. GDS2022 ran from November 2021 to March 2022 and took 15–60 min to complete (depending on drug use). GDS2022 was translated into 11 languages (Danish, Dutch, English, Finnish, French, German, Hungarian, Italian, Portuguese, Romanian and Spanish). Capturing a purposive sample of people who use recreational drugs, recruitment into GDS is facilitated by mainstream and social media and harm reduction organisations; see Winstock et al. [24] for further details on recruitment and methods. GDS is a non‐probability survey but nonetheless has been demonstrated to recruit people who use alcohol and cannabis who are similar in age and gender to people completing general household surveys [25]. GDS received ethics approval from University College London (11671/001), which was registered at RMIT University (2020‐23913‐11758) and The University of Queensland (2017001452).

### 2.2 Sample

The sample for this study was limited to those respondents who provided a valid answer to the first question of the NoLo section of the survey.

### 2.3 Measures

Survey items are presented in Appendix A.

#### 2.3.1 Outcome measure—NoLo use

The NoLo section was presented to all respondents regardless of alcohol or other drug consumption history:

Have you ever consumed any NoLo products? These include beers, ciders, spirits or wines that are: Low alcohol products—not more than 1.2% ABV; De‐alcoholised—not more than 0.5% ABV; Alcohol‐free—usually no more than 0.05%. Response options were No/Yes but not in the last 12 months/Yes in the last 12 months.

Respondents were informed “NoLo does not refer to other kinds of drinks with no or a trace amount of alcohol—such as coffees, teas, fruit juices and soft drinks.”

Those indicating NoLo use were presented with items about NoLo beliefs and motives for using NoLo products. Items were created by the study team using previous literature and linked to COM‐B components [26]. Questions probing reasons for not using NoLo were presented to respondents who ticked no to the first NoLo item.

#### 2.3.2 Alcohol consumption

As part of the drug screen respondents were asked when they last used alcohol. Response options were: Never/in the last 30 days/between 31 days and 12 months ago/more than 12 months ago. Respondents consuming alcohol in the last year completed the Alcohol Use Disorders Identification Test (AUDIT) [27], a 10‐item questionnaire used to assess risk of alcohol dependence. Scores ranged from 1 to 40 were categorised as lower risk (1–7), increasing risk (8–15), higher risk (16–19) and possible alcohol dependence (20+). Respondents who reported they did not use alcohol in the last year did not see the AUDIT questions.

#### 2.3.3 Sociodemographic measures

GDS2022 also contained a broad range of demographic measures but for the purpose of this study we included gender, age, ethnicity, employment status and country of residence (see Appendix A).

### 2.4 Data analysis

To address RQ1, we used descriptive statistics and chi square tests of association to examine use of NoLo products by age, gender, employment status, ethnicity and alcohol use. Multi‐level, random intercept logistic regression models were used to explore demographic and alcohol consumption factors associated with NoLo use. These analyses involved clustering for country of residence, which was entered as a random factor to account for confounders relating to country that were not incorporated into the model such as drinking culture, legal age of drinking, taxation or affordability. Age was rescaled in 5‐year increments (the quadratic term was also entered) and fixed factors were gender, drinker status (non‐drinker—with AUDIT score = 0, compared to respondents in the four AUDIT categories) employment status and ethnicity. To address RQ2, differences in beliefs and reasons for using and not using NoLo products were examined using descriptive statistics and chi square tests of association. To address RQ3, beliefs, reasons for using/not using NoLos were mapped to the COM‐B model by two authors experienced in using the COM‐B model, and identified as possible enablers or barriers to NoLo use for increasing, higher and possibly dependent respondents (referred to hereafter as high‐risk drinking respondents). See Appendix B. We used pairwise deletion to deal with missing data.

## 3 RESULTS

### 3.1 Sample

The sample included 30,033 respondents from 22 countries (see Table S1) who answered the NoLo screener. Notably, a large proportion of the respondents were from Germany (*N* = 12,183; 40.6%), in line with other GDS surveys [28]. While country comparisons are not the focus of this paper, respondents from Europe (Poland, Netherlands, Germany, Switzerland and Sweden) were more likely to report last 12‐month NoLo use. Over half (57.0%) were low risk drinkers by AUDIT category. The sample consisted predominantly of cis men (59.5%), and the majority (88.0%) reported alcohol use in the last year (Table 1).

Do demographic factors differ across NoLo product use?

**TABLE 1:** Bivariate relationships between demographic characteristics/alcohol variables and NoLo product use categories.

	Whole sample	Never used NoLo, *N* (%)	Used NoLo >12 months ago, *N* (%)	Used NoLo <12 months ago, *N* (%)	Chi square *χ* ^2^ (DF), *p* value, effect size
Whole sample	*N* = 30,033	9491 (31.6)	4855 (16.2)	15,687 (52.2)	
Gender					*χ* ^2^ = 35.59 (4), *p* < 0.001, *V* = 0.024
Cis man	17,857 (59.5)	5617 (59.2)^a^	2885 (59.4)^a,b^	9355 (59.6)^a^	
Cis woman	11,206 (37.3)	3489 (36.8)^a^	1808 (37.2)^a^	5909 (37.7)^a^	
Trans/non‐binary/other	970 (3.2)	385 (4.1)^a^	162 (3.3)^a,b^	423 (2.7)^b^	
Age Mdn = 34, IQR = 26–45					*χ* ^2^ = 275.65 (6), *p* < 0.001, *V* = 0.068
16–25	7129 (23.7)	2674 (28.2)^a^	913 (18.8)^b^	3542 (22.6)^c^	
26–35	9221 (30.7)	2680 (28.2)^a^	1358 (28.0)^a^	5183 (33.0)^b^	
36–45	6228 (20.7)	1861 (19.6)^a^	1142 (23.5)^b^	3225 (20.6)^a^	
46+	7455 (24.8)	2276 (24.0)^a^	1442 (29.7)^b^	3737 (23.8)^a^	
Alc last year					***χ*** ^**2**^ **= 508.89 (2), *p* < 0.001, *V* = 0.130**
No	3618 (12.0)	1470 (15.5)^a^	880 (18.1)^b^	1267 (8.1)^c^	
Yes	26,415 (88.0)	8021 (84.5)^a^	3975 (81.9)^b^	14,420 (91.9)^c^	
AUDIT (*N* = 26,374)					*χ* ^2^ = 29.09 (6), *p* < 0.001, *V* = 0.023
Low risk (1–7)	15,028 (57.0)	4545 (56.7)^a^	2326 (58.6)^a^	8157 (56.7)^a^	
Increasing risk (8–15)	8430 (32.0)	2517 (31.4)^a,b^	1177 (29.7)^b^	4736 (32.9)^a^	
Higher risk (16–19)	1574 (6.0)	489 (6.1)^a^	244 (6.2)^a^	841 (5.8)^a^	
Possible dependence (20+)	1342 (5.1)	460 (5.7)^a^	219 (5.5)^a,b^	663 (4.6)^b^	
Missing	41				
Ethnicity (*N* = 29,745)					*χ* ^2^ = 95.30 (2), *p* < 0.001, *V* = 0.057
White	25,561 (85.9)	7804 (83.4)^a^	4093 (85.0)^a^	13,664 (87.7)^b^	
Other ethnicity	4184 (14.1)	1552 (16.6)^a^	724 (15.0)^a^	1908 (12.3)^b^	
Missing	288				
Employment status (*N* = 30,017)					*χ* ^2^ = 146.18 (4), *p* < 0.001, *V* = 0.049
Full time	16,744 (55.8)	5068 (53.4)^a^	2610 (53.8)^a^	9066 (57.8)^b^	
Part‐time	5604 (18.7)	1637 (17.3)^a^	918 (18.9)^b^	3049 (19.4)^b^	
Not‐working	7669 (25.5)	2777 (29.3)^a^	1327 (27.3)^b^	3565 (22.7)^c^	
Missing	16				

*Note*: Each superscript letter denotes a subset of each demographic characteristic/alcohol category (rows) that do not differ significantly from each other at the 0.05 level—for example—for the alc last year variable each column differs significantly, meaning that there is an association between drinking alcohol in the last year and NoLo use. Results in bold indicate medium or larger effect sizes.

Abbreviations: AUDIT, Alcohol Use Disorders Identification Test; IQR, interquartile range; NoLo, no and low alcohol.

Just over half of the sample (*N* = 15,687; 55.2%) reported using a NoLo product in the last 12 months, and 16.2% (*N* = 4855) had used NoLo products but not in the last 12 months (Table 1). Gender was significantly associated with NoLo product use. Trans/non‐binary respondents were less likely to report NoLo use in the last 12 months compared to never use. Age was significantly associated with NoLo product use. Respondents aged over 36 were more likely to report NoLo use more than 12 months ago compared to recent use. Respondents who had consumed alcohol in the last year were more likely to report NoLo use in the last year than less recently or not at all. Respondents at higher risk of alcohol dependency were less likely to report NoLo use in the last 12 months compared to never using NoLo products. White respondents and employed respondents were more likely to report NoLo use in the last 12 months compared to never use or use more than 12 months ago. Table S2 displays demographic characteristics in each NoLo use category by drinker status.

Table 2 presents the results of two sets of multivariable binary regression models, clustering for country. Model 1 compares ever versus never use of NoLo products. Wald tests indicated that age, drinker status and employment were significant predictors. As the age of respondents increases the odds of reporting ever consuming NoLo the beverages are constantly increasing. However, this rate of increase in reporting NoLo beverage consumption slows down for older respondents. This non‐linear relationship suggests that while older age is associated with higher odds of the outcome, the association diminishes at higher ages (Figure S1). Compared to non‐drinkers, respondents in all AUDIT categories were more likely to report NoLo use. As AUDIT score increases OR typically increases until AUDIT 20+ when OR is less than AUDIT 16–19. Compared to those in full time employment, part‐time and not working respondents were less likely to report NoLo use. Model 2 compares respondents who report recent NoLo use with those having used these products more than 12 months ago. Wald tests indicated that age gender, drinker status and employment were significant predictors. As age increases, the odds of respondents consuming NoLo products in the last year decreases, up until around 47 years of age when it increases (Figure S2). Compared to trans/non‐binary respondents, cis respondents were more likely to report using NoLo products in the last 12 months. Similar to model 1, compared to non‐drinkers, respondents in all AUDIT categories were more likely to report NoLo use in the last 12 months. Also similar to model 1, compared to those in full time employment, part time working and not working respondents were less likely to report NoLo use in the last 12 months.

Do beliefs about NoLo products and reason for their use/non‐use differ by level of risky drinking?

**TABLE 2:** Multi level logistic regression models with country included as a random effect.

Variable	Wald‐test *χ* ^2^, *p*	Model 1—ever versus never, *N* = 29,692			Wald‐test *χ* ^2^, *p*	Model 2—last 12 months versus more than 12 months ago, *N* = 20,351		
		OR	95% CI	*p*		OR	95% CI	*p*
Age	137.73, *p* < 0.001				88.87, *p* < 0.001			
Age^5^		1.234	1.165–1.306	< 0.001		0.715	0.661–0.774	< 0.001
Age^25^		0.991	0.987–0.994	< 0.001		1.018	1.013–1.022	< 0.001
Gender	6.00, *p* = 0.050				8.34, *p* = 0.015			
Cis man		1.044	0.905–1.204	0.557		1.061	0.872–1.293	0.553
Cis woman		1.111	0.962–1.284	0.152		1.172	0.960–1.430	0.119
Drinker status	132.38, *p* < 0.001				300.35, *p* < 0.001			
AUDIT 1–7		1.494	1.380–1.616	< 0.001		2.232	2.019–2.469	< 0.001
AUDIT 8–15		1.625	1.490–1.772	< 0.001		2.535	2.266–2.834	< 0.001
AUDIT 16–19		1.634	1.432–1.866	< 0.001		2.250	1.895–2.670	< 0.001
AUDIT 20+		1.464	1.275–1.681	< 0.001		2.128	1.774–2.552	< 0.001
Employment	23.09, *p* < 0.001				67.87, *p* < 0.001			
Part‐time		1.060	0.987–1.139	0.108		0.823	0.751–0.901	< 0.001
Not working		0.880	0.823–0.942	< 0.001		0.691	0.631–0.755	< 0.001
Ethnicity	2.09, *p* = 0.148				0.12, *p* = 0.726			
White		1.065	0.978–1.160	0.148		1.021	0.908–1.148	0.727
REvar		0.303				0.181		
ICC		0.084				0.052		

*Note*: Table presents odds ratios, confidence intervals and significance of the variables associated with NoLo product use. Number of groups = 21. Reference categories: gender = non‐binary, drinker status = non‐drinker; employment = full time; ethnicity = other than White. For categorical variables p value relates to the robust (omnibus) test.

Abbreviations: AUDIT, Alcohol Use Disorders Identification Test; CI, confidence interval; ICC, intra‐class correlation; NoLo, no and low alcohol; OR, odds ratio; REvar, random effect variance.

The most strongly endorsed belief was that NoLo products are healthier than alcoholic drinks (*N* = 13,331; 45.2%). The least endorsed belief was that NoLo products can influence someone to drink more regularly (*N* = 1463; 5.0%). Higher risk and possible dependence respondents were the most likely to endorse that ‘NoLo products can help me to drink less alcohol’. Higher risk drinking respondents were also more likely to endorse the idea that NoLo products were useful for when they wanted to pretend they were drinking, and were lower in calories, than respondents who did not drink or were low risk drinkers. Respondents in the possible dependence category were more likely than others to believe NoLo products would influence them to drink more regularly (Table 3).

**TABLE 3:** Beliefs about NoLo products in the sample and by Alcohol Use Disorders Identification Test category.

NoLo product beliefs	Whole sample, *N* = 29,508 (%)	Not drank in last 12 months, *N* = 3565	Low risk, *N* = 14,793	Increasing risk, *N* = 8257	Higher risk, *N* = 1540	Possible dependence, *N* = 1315	Chi square *χ* ^2^ (DF), *p* value, effect size
Are healthier than alcoholic drinks	13,331 (45.2)	1335 (37.4)^a^	6352 (42.9%)^b^	4151 (50.3)^c^	798 (51.8%)^c^	680 (51.7)^c^	*χ* ^2^ = 251.77 (4), *p* < 0.001, *V* = 0.092
**Can help me to drink less alcohol**	**10,301 (34.9)**	**795 (22.3)** ^**a**^	**4606 (31.1)** ^**b**^	**3552 (43.0)** ^**c**^	**722 (46.9)** ^**d**^	**616 (46.8)** ^**d**^	***χ*** ^**2**^ **= 759.55 (4), *p* < 0.001, *V* = 0.161**
Make it fashionable to say no to alcohol	7062 (23.9)	820 (23)^a,b,c,d^	3588 (24.3)^c,d^	2022 (24.5)^b,d^	327 (21.2)^a^	298 (22.7)^a,b,c,d^	*χ* ^2^ = 11.19 (4), *p* = 0.024, *V* = 0.019
Just another way for the alcohol industry to make money	7033 (23.8)	891 (25.0)^a^	3406 (23)^b^	2007 (24.3)^a^	378 (24.5)^a,b^	347 (26.4)^a^	*χ* ^2^ = 14.23 (4), *p* = 0.007, *V* = 0.022
Significantly lower in calories	5782 (19.6)	422 (11.8)^a^	2774 (18.8)^b^	1901 (23)^c^	359 (23.3)^c^	319 (24.3)^c^	*χ* ^2^ = 235.59 (4), *p* < 0.001, *V* = 0.089
Are useful for when I want to pretend I am drinking	5371 (18.2)	561 (15.7)^a^	2502 (16.9)^a^	1650 (20)^b^	335 (21.8)^b,c^	314 (23.9)^c^	*χ* ^2^ = 90.00 (4), *p* < 0.001, *V* = 0.055
Can influence me to drink alcohol more regularly	1463 (5.0)	217 (6.1)^a^	658 (4.4)^b^	402 (4.9)^b^	84 (5.5)^a,b^	101 (7.7)^c^	*χ* ^2^ = 39.49 (4), *p* < 0.001, *V* = 0.037

*Note*: Each superscript letter denotes a subset of drinker categories (rows) that do not differ significantly from each other at the 0.05 level. Results in bold indicate medium or larger effect sizes.

Abbreviation: NoLo, no and low alcohol.

The most common motive for using NoLo products was to avoid getting drunk (*N* = 9404; 46.8%; see Table 4). The least endorsed motive was that it was fashionable (*N* = 259; 1.3%). Higher risk drinking respondents were more likely to use NoLo products to drink alcohol less often and avoid embarrassing situations, but less likely to endorse the statement about staying safe when driving, and that they liked the taste of NoLo products when compared to non‐drinking and low risk drinking respondents.

**TABLE 4:** Reasons for using NoLo products by Alcohol Use Disorders Identification Test category.

Reasons using no/no products	Whole sample, *N* = 20,100 (%)	Not drank in last 12 months, *N* = 2100	Low risk, *N* = 10,288	Increasing risk, *N* = 5765	Higher risk, *N* = 1057	Possible dependence, *N* = 858	Chi square *χ* ^2^ (DF), *p* value, effect size
To avoid getting drunk	9404 (46.8)	897 (42.7)^a^	4675 (45.4)^b^	2866 (49.7)^c^	525 (49.7)^c^	430 (50.1)^c^	*χ* ^2^ = 48.65 (4), *p* < 0.001, *V* = 0.049
**To stay safe when I am driving**	**8026 (39.9)**	**566 (27.0)** ^**a**^	**4368 (42.5)** ^**b**^	**2438 (42.3)** ^**b**^	**373 (35.3)** ^**c**^	**271 (31.6)** ^**c**^	***χ*** ^**2**^ **= 222.61 (4), *p* < 0.001, *V* = 0.105**
**I like the taste of NoLo**	**5803 (28.9)**	**520 (24.8)** ^**a**^	**3358 (32.6)** ^**b**^	**1587 (27.5)** ^**c**^	**198 (18.7)** ^**d**^	**132 (15.4)** ^**d**^	***χ*** ^**2**^ **= 222.37 (4), *p* < 0.001, *V* = 0.105**
To look after mental/physical health	5424 (27.0)	682 (32.5)^a^	2610 (25.4)^b^	1562 (27.1)^c^	309 (29.2)^a,c^	255 (29.7)^a,c^	*χ* ^2^ = 51.76 (4), *p* < 0.001, *V* = 0.051
**I am trying to drink less often**	**3361 (16.7)**	**224 (10.7)** ^**a**^	**1022 (9.9)** ^**a**^	**1398 (24.2)** ^**b**^	**360 (34.1)** ^**c**^	**353 (41.1)** ^**d**^	***χ*** ^**2**^ **= 1225.50 (4), *p* < 0.001, *V* = 0.247**
To help me consume fewer calories	2832 (14.1)	185 (8.8)^a^	1506 (14.6)^b^	861 (14.9)^b^	159 (15.0)^b^	117 (13.6)^b^	*χ* ^2^ = 55.26 (4), *p* < 0.001, *V* = 0.052
To fit in better with others who are drinking	1626 (8.1)	196 (9.3)^a^	780 (7.6)^b^	462 (8.0)^a,b^	103 (9.7)^a^	85 (9.9)^a^	*χ* ^2^ = 15.66 (4), *p* = 0.004, *V* = 0.028
**To avoid doing something embarrassing when drunk**	**1566 (7.8)**	**215 (10.2)** ^**a**^	**569 (5.5)** ^**b**^	**494 (8.6)c**	**141 (13.3)** ^**d**^	**146 (17.0)** ^**e**^	***χ*** ^**2**^ **= 242.25 (4), *p* < 0.001, *V* = 0.110**
To avoid interactions with other drugs	1323 (4.4)	153 (7.3)^a^	624 (6.1)^b^	400 (6.9)^a^	71 (6.7)^a,b^	72 (8.4)^a^	*χ* ^2^ = 11.96 (4), *p* = 0.018, *V* = 0.024
Because I/my partner is pregnant	633 (3.1)	65 (3.1)^a,b^	395 (3.8)^b^	146 (2.5)^a,c^	12 (1.1)^d^	14 (1.6)^c,d^	*χ* ^2^ = 43.81 (4), *p* < 0.001, *V* = 0.047
My friends family like me to drink NoLo	508 (2.5)	40 (1.9)^a^	183 (1.8)^a^	179 (3.1)^b^	47 (4.4)^c^	59 (6.9)^d^	*χ* ^2^ = 116.01 (4), *p* < 0.001, *V* = 0.076
It is fashionable to drink NoLo	259 (1.3)	27 (1.3)^a^	136 (1.3)^a^	74 (1.3)^a^	11 (1.0)^a^	11 (1.3)^a^	*χ* ^2^ = 0.60 (4), *p* = 0.963, *V* = 0.005

*Note*: Each superscript letter denotes a subset of drinker categories (rows) that do not differ significantly from each other at the 0.05 level. Results in bold indicate medium or larger effect sizes.

Abbreviation: NoLo, no and low alcohol.

The most commonly endorsed reason for not using NoLo products was the statement ‘I drink alcohol for the effect and so they offer me nothing’ (*N* = 4204; 44.7%; Table 5). Respondents in the higher risk and possible dependence AUDIT categories were more likely to endorse the statement than other respondents—78.6% of those in the possible dependence category compared with 38.1% of those in the low‐risk category. Similarly, respondents in the higher risk and possible dependence AUDIT categories were more likely to endorse the statement relating to not having a good time when drinking NoLo products than other respondents, as well as the statement about the expense of NoLo products.

What COM‐B factors could be targeted in order to encourage higher risk drinkers to drink NoLo products in place of regular strength products?

**TABLE 5:** Reasons for not using no and low alcohol products by Alcohol Use Disorders Identification Test category.

Reasons for not using no/no products	Whole sample, *N* = 9407 (%)	Not drank in last 12 months, *N* = 1462	Low risk, *N* = 4505	Increasing risk, *N* = 2491	Higher risk, *N* = 483	Possible dependence, *N* = 457	Chi square *χ* ^2^ (DF), *p* value, effect size
**I drink for the effect so they offer me nothing**	**4204 (44.7)**	**190 (13.3)** ^**a**^	**1717 (38.1)** ^**b**^	**1569 (63.0)** ^**c**^	**367 (76.0)** ^**d**^	**359 (78.6)** ^**d**^	***χ*** ^**2**^ **= 1413.61 (4), *p* < 0.001, *V* = 0.388**
**I prefer to stick to water or soft drinks**	**4167 (44.3)**	**769 (52.6)** ^**a**^	**2076 (46.1)** ^**b**^	**997 (40.0)** ^**c**^	**175 (36.2)** ^**c,d**^	**148 (32.4)** ^**d**^	***χ*** ^**2**^ **= 104.09 (4), *p* < 0.001, *V* = 0.105**
Never occurred to me to try them	2849 (30.3)	388 (26.5)^a^	1502 (33.3)^b^	708 (28.4)^a^	123 (25.5)^a^	126 (27.6)^a^	*χ* ^2^ = 40.64 (4), *p* < 0.001, *V* = 0.066
I have not heard of them	1648 (17.5)	212 (14.5)^a^	841 (18.7)^b^	452 (18.1)^b^	68 (14.1)^a^	74 (16.2)^a,b^	*χ* ^2^ = 18.52 (4), *p* < 0.001, *V* = 0.044
I do not like the way they taste	1423 (15.1)	228 (15.6)^a.b^	651 (14.5)^b^	406 (16.3)^a^	78 (16.1)^a,b^	58 (12.7)^a,b^	*χ* ^2^ = 7.03 (4), *p* = 0.135, *V* = 0.027
**Too expensive for what they are**	**830 (8.8)**	**56 (3.8)** ^**a**^	**328 (7.3)** ^**b**^	**299 (12.0)** ^**c**^	**75 (15.5)** ^**d**^	**72 (15.8)** ^**d**^	***χ*** ^**2**^ **= 144.10 (4), *p* < 0.001, *V* = 124**
They are not widely available in my area	628 (6.7)	36 (2.5)^a^	317 (7.0)^b^	199 (8.0)^b^	42 (8.7)^b^	34 (7.4)^b^	*χ* ^2^ = 53.04 (4), *p* < 0.001, *V* = 0.075
**I wouldn't have a good time**	**591 (6.3)**	**71 (4.9)** ^**a**^	**148 (3.3)** ^**b**^	**226 (9.1)** ^**c**^	**67 (13.9)** ^**d**^	**79 (17.3)** ^**d**^	***χ*** ^**2**^ **= 247.74 (4), *p* < 0.001, *V* = 0.162**
**Friends/family prefer me to drink alcoholic drinks**	**184 (2.0)**	**10 (0.7)** ^**a**^	**52 (1.2)** ^**a**^	**71 (2.9)** ^**b**^	**21 (4.3)** ^**b,c**^	**29 (6.3)** ^**c**^	***χ*** ^**2**^ **= 98.58 (4), *p* < 0.001, *V* = 0.102**

*Note*: Each superscript letter denotes a subset of drinker categories (rows) that do not differ significantly from each other at the 0.05 level. Results in bold indicate medium or larger effect sizes.

Table 6 displays findings mapped onto the COM‐B model. Table 6 shows items as important (indicated by ‘yes’) when high‐risk drinking respondents were more likely or less likely to endorse the statement than other respondents. The expense of NoLo products was a physical opportunity barrier to their use. Social opportunity could be a barrier and an enabler to NoLo use. Reflective motivation items relating to beliefs about the health effects of NoLo use were already strongly endorsed by high‐risk drinking respondents. Automatic motivational items showed a distinct difference between high and lower risk drinking and non‐drinking respondents. Table 6 illustrates which parts of the TDF could be targeted to overcome the barriers and support the enablers to NoLo use for higher risk drinking respondents. TDF components can aid intervention developers to understand possible mechanisms that can bring about change. These include environmental context and resources, social influences and reinforcement and emotion.

**TABLE 6:** Using the capability, opportunity, motivation, behaviour (COM‐B) and theoretical domains framework (TDF) to explore how to target behaviour of NoLo use in place of regular strength alcohol in higher risk drinking respondents.

COM‐B component	Survey enablers (no or yes indicates whether higher risk drinkers endorsed the item differently to other respondents and/or if proportions of higher risk respondents endorsing the item indicated that it could be a potential target for behaviour change)	Survey barriers (no or yes indicates whether higher risk drinkers endorsed the item differently to other respondents and/or if proportions of higher risk respondents endorsing the item indicated that it could be a potential target for behaviour change)	Summary	TDF domain
Physical capability	N/A	N/A	N/A	N/A
Psychological capability	They are healthier: yes	Not occurred to me to try them: yes Not heard of them: no	Higher risk drinking respondents already have sufficient knowledge about possible benefits of NoLo, but may not have considered trying these products.	Knowledge Skills
Physical opportunity		Not widely available: no Too expensive: yes	Available to higher risk drinking respondents but seen as too expensive by some.	Environmental context and resources
Social opportunity	Useful to pretend when drinking: yes Make it fashionable to say no: no Friends/family like me to drink NoLo: no Fit in better with others: yes Fashionable to drink: no	Friends family prefer me to drink alcohol: yes	Although some items only endorsed by small proportion, beliefs about others' views are more important influence on higher risk drinking respondents than other respondents.	Social influences
Reflective motivation	Help me to drink less alcohol: yes They are lower in calories: yes To look after mental/physical health: yes Avoid interaction with other drugs: no Help consume fewer calories: yes Trying to drink less often: yes Stay safe when driving: no Pregnancy: no		Nearly half of higher risk drinking respondents believe NoLo products can help them to drink less often and a third believe NoLo use can help physical/mental health.	Beliefs about capabilities Goals Beliefs about consequences
Automatic motivation	Avoid getting drunk: yes Avoid something embarrassing: yes Like the taste: no	Influence me to drink more regularly: yes Another way for the industry to make money: no Drink for the effect so they offer me nothing: yes Don't like the taste: no Prefer to stick to water/other soft drinks: yes Would not have a good time: yes	While higher risk drinkers may be motivated to avoid the consequences of drinking, they enjoy being intoxicated and may be cued to consume more alcohol products by the similarity of NoLo brands. They are less likely to say they want to stick to other soft drinks and more likely to believe they would not have a good time when consuming NoLos products.	Reinforcement Emotion

*Note*: TDF domains indicate the areas where higher risk drinking respondents could be targeted to increase NoLo use.

Abbreviations: N/A, not applicble; NoLo, no and low alcohol.

## 4 DISCUSSION

This study extends previous research by exploring beliefs about NoLo products and motives for their use by level of risky drinking. In a large international sample, two‐thirds of respondents had consumed NoLo products—more than half in the last 12 months. Controlling for all variables, the demographic factors associated with recent NoLo use were age, and employment status. There was a non‐linear relationship between NoLo use and age. While NoLo use increased with age, there appeared to be a turning point in middle age when the odds of reporting NoLo use diminished. This may indicate that for older drinkers, who could have more entrenched alcohol consumption habits, policies encouraging NoLo use may not be acceptable, although they could have the potential to reduce harms. Employment can be a useful proxy for SES and in this study, those who were employed were more likely to have used NoLo products than those who were not working supporting previous research suggesting NoLo use is more prevalent in affluent consumers [13, 14]. NoLo use was more prevalent amongst respondents who reported consuming alcohol, compared to respondents who did not drink alcohol. There was also a non‐linear relationship between NoLo use and AUDIT category. NoLo use was more prevalent in respondents in the higher risk (AUDIT 16–19) category, who may be at a point where they are considering making changes to their drinking behaviours, compared to those scoring 20+, for whom change may be more difficult if experiencing dependence. Also striking was that high‐risk drinking respondents chose not to consume NoLo products because they wanted to feel intoxicated and would not have a good time, thus NoLo products were unable to fulfil their drinking motives.

Importantly, findings suggest that NoLo products may be part of a strategy used by high‐risk drinking respondents to control alcohol intake and avoid unwanted social consequences of consuming too much alcohol. Such short term outcomes are often identified as important motivators for reducing alcohol consumption [29]. Equally important, respondents in the possible dependence category were more likely to believe NoLo products would make them drink more regularly, suggesting these products might encourage further alcohol consumption in some individuals.

Barriers to NoLo use were conceptualised using the COM‐B model. Physical opportunity barriers included the cost of NoLo products. Social opportunity in the form of friends/family expectations could be both a barrier and enabler to NoLo use. However, social support is often overlooked in individual level interventions. A recent review has shown that targeting social factors is one of the most effective ways to bring about behaviour change [30]. Higher risk drinking respondents thought NoLo products could help them drink less, but they also felt that using them might mean losing the enjoyment of drinking. Emotional and intoxication‐related barriers also affected their motivation. However, avoiding the negative consequences of drinking could be an enabler for increased NoLo use. For example, repeated NoLo use could result in fewer regrets from the drinking occasion – although research shows that higher risk drinkers have fewer regrets than lower risk drinkers [31].

It is useful to consider these findings through the lens of drinking motives, due to their prevalence and utility in explaining and changing drinking behaviours [20] and because COM‐B as a meta‐theory can be usefully extended with behaviour specific theory when developing intervention approaches [32]. A key takeaway from this analysis is that people who are drinking for enhancement motives (e.g., to feel intoxicated) may be less amenable to substituting regular strength alcohol products for NoLo products. There are also indications that those drinking primarily for social motives (e.g., to enjoy social events and celebrations) or conformity motives (e.g., to fit in) could be amenable to change, due to endorsement of items relating to social opportunity. Our findings leave some unanswered questions about whether NoLo substitution would be acceptable for those who drink for coping motives (e.g., to alleviate poor mood), particularly due to the relationship between alcohol consumption and mental health [33]. Further research should explore how drinking motives interact with the acceptability of alcohol harm reduction strategies, such as NoLo use.

### 4.1 Limitations

Limitations include the opportunistic recruitment methods and cross‐sectional design, which means we make no claims for the representativeness of the findings. While we included a definition of NoLo products at the start of that survey section, it is important to note that the UK uses a more narrow definition of NoLo products than other jurisdictions [5], and other research on this topic has used different definitions [34]. We also did not explore whether the relative price of NoLo products and alcohol products influenced NoLo use.

### 4.2 Implications

The COM‐B framework applied to this topic will be useful for those who wish to develop interventions aimed at increasing substitution behaviours in higher risk drinkers. In particular, such efforts should focus on the cost/accessibility of NoLo products, social influence and beliefs about the purpose of alcohol. Conversely, higher risk drinkers may be primed to drink regular strength products by increased NoLo product visibility and accessibility, due to the strong similarities in branding [16]. As our findings highlight, higher risk drinkers are motivated by beliefs about intoxication and enjoyment when drinking, which cannot be replaced by a NoLo alternative. Although the previous UK government expressed a desire for increased substitution of NoLo products in higher risk drinkers, it may be that this strategy unintentionally reinforces alcohol industry messaging, for example about individual responsibility [35]. Notably, a quarter of respondents endorsed the statement that NoLo products were another way for the alcohol industry to make money. Research, therefore, should focus on the interaction between drinking motives and acceptability of NoLo substitution, while furthering our understanding of how to replace the psychoactive elements of alcohol consumption.

## 5 CONCLUSIONS

Higher risk alcohol drinking respondents were drinking alcohol for its effects and NoLo products cannot replace this feeling. While targeting higher risk drinkers to swap regular products for NoLo substitutes may be part of a broader approach to reducing alcohol harms, this should not be seen as a panacea. Intervention efforts should focus on promoting alcohol‐free novel experiences and viable activities that could replace the positive reinforcing effects of alcohol, leading to longer‐term culture change.

## AUTHOR CONTRIBUTIONS

Each author certifies that their contribution to this work meets the standards of the International Committee of Medical Journal Editors.

## CONFLICT OF INTEREST STATEMENT

The authors have no interests to declare.

## Supporting information


**FIGURE S1:** Impact of age on ever use of no and low alcohol products compared to never use, controlling for other variables in model 1—Table 2.
**FIGURE S2:** Impact of age on use of no and low alcohol products in the last 12 months compare to ever use of no and low alcohol products, controlling for other variables in model 1—Table 2.
**TABLE S1:** NoLo use by respondent country.
**TABLE S2:** Demographic factors across drinker categories relative to NoLo use.

## Data Availability

The data that support the findings of this study are available from the corresponding author upon reasonable request.

## APPENDIX A

**Survey items from Global Drug Survey 2024 used in the current paper**

**Demographics**

**Age**

How old are you? From 16 to <85

**Gender**

Gender and sex were measured through two items which were then combined to make a composite variable:

How do you describe your gender? Gender refers to current gender, which may be different to sex recorded at birth and may be different to what is indicated on legal documents.

- Man or male
- Woman or female
- Non‐binary
- I use a different term (please specify)

What was your sex recorded at birth?

- Male
- Female
- Another term (please specify)

The variable used in the analysis contains the following categories

- Cis‐woman
- Cis‐man
- Trans man
- Trans woman
- Non‐binary
- Other gender identity

It is compiled using the following formula

Cis‐woman = Female gender, assigned female at birth

Cis‐man = Male gender, assigned male at birth

Trans man = Male gender, assigned female at birth

Trans woman‐ Female gender, assigned male at birth

Non‐binary = non‐binary

Other gender ID = any other gender ID or other different identity

**Which country do you live in?**

Respondents selected their current country of residence.

**What is your ethnicity?**

White

Black/African American

Asian

Hispanic/Latino

Aboriginal/Maori

Native American

Mixed

Other—please specify

For this study we compared White with all other ethnicities.

**Are you currently in paid employment?**

Yes (full‐time)

Yes (part‐time < 35 h/week)

No (looking for work)

No (retired)

No (undertaking home duties)

No (A non‐working student).

No (Permanently ill or unable to work).

No (none of the above)

For this study we compared full time, part time and combined all the ‘no’ responses.

**Alcohol use in the last 12 months**

The Global Drug Survey drug screen contains a list of substances including alcohol.

When did you last use the following drugs?

Never

In the last 30 days

Between 31 days and 12 months ago

More than 12 months ago

Those who had used alcohol in the last 12 months were coded as 1 and those reporting never using alcohol or using it more than 12 months ago were coded as 0.

**Respondents who reported alcohol use in the last 12 months were presented with the Alcohol Use Disorders Identification Test**

How often do you have a drink containing **alcohol**?

Monthly or less

2–4 times per month

2–3 times a week

4 or more times a week

How many standard drinks do you have on a day when you drink?

1 or 2

3 or 4

5 or 6

7 to 9

10 or more

How often do you have 6 or more drinks on one occasion?

Never

Less than monthly

Monthly

Weekly

Daily/almost daily

How often during the last year have you found that you were not able to stop drinking once you had started?

Never

Less than monthly

Monthly

Weekly

Daily/almost daily

How often during the last year have you failed to do what was normally expected of you because of drinking?

Never

Less than monthly

Monthly

Weekly

Daily/almost daily

How often during the last year have you needed a drink in the morning to get yourself going after a heavy drinking session?

Never

Less than monthly

Monthly

Weekly

Daily/almost daily

How often during the last year have you had a feeling of guilt or remorse after drinking?

Never

Less than monthly

Monthly

Weekly

Daily/almost daily

How often during the last year have you been unable to remember what happened the night before because you had been drinking?

Never

Less than monthly

Monthly

Weekly

Daily/almost daily

Have you or someone else been injured as a result of your drinking?

No

Yes, but not in the last year

Yes, during the last year

Has a friend, relative, doctor, or other health worker been concerned about your drinking or suggested you cut down?

No

Yes, but not in the last year

Yes, during the last year

AUDIT items are scored 0–4 other than the final two which are score 0 = never, 2 = yes but not in the last year, 4 = yes during the last year

**NoLo section**

Have you ever consumed any NoLo products?

These include beers, ciders, spirits or wines that are:

Low alcohol products—not more than 1.2% ABV

De‐alcoholised—not more than 0.5% ABV

Alcohol‐free—usually no more than 0.05%

No

Yes, but not in the last 12 months

Yes, during the last 12 months

When you think about NoLo products, which of the following statements do you agree with (if any?) Check all that apply

- NoLo products are healthier than alcoholic drinks
- NoLo products can help me to drink less alcohol
- NoLo products can influence me to drink alcohol more regularly
- NoLo products are useful for when I want to pretend I am drinking alcohol
- NoLo products make it more fashionable to say no to alcohol
- NoLo products are significantly lower in calories
- NoLo products are just another way for the alcohol industry to make money

For which of the following reasons do you drink NoLo products? Select all that apply.

- To look after my mental/physical health
- To avoid getting drunk
- To avoid interactions with other drugs
- To help me consume fewer calories
- I am trying to drink less often
- To avoid doing something embarrassing when drunk
- To stay safe when I am driving
- My friends/family like me to drink NoLo products
- To fit in better with others who are drinking
- It is fashionable to drink NoLo products
- I like the taste of NoLo products
- Because I am pregnant/my partner is pregnant, or I want to become pregnant

What are the reasons why you have not consumed a NoLo product? Select all that apply.

- I drink alcohol for the effect, so these offer me nothing of interest
- They are not widely available in my area
- I have not heard of these products
- I do not like the way they taste
- Never occurred to me to try these products
- I prefer to stick to water or soft drinks (e.g., soda, juice)
- I wouldn't have a good time
- They are too expensive for what they are
- My friends/family prefer me to drink alcoholic products

## APPENDIX B

**Table:**

Item	Possible mechanism of action (TDF domain)	Endorsement indicates an enabler or barrier?	COM‐B component
NoLo products are healthier than alcoholic drinks	Knowledge	Enabler	Psychological capability
NoLo products can help me to drink less alcohol	Beliefs about capabilities	Enabler	Reflective motivation
NoLo products can influence me to drink alcohol more regularly	Reinforcement	Barrier	Automatic motivation
NoLo products are useful for when I want to pretend I am drinking alcohol	Social influences	Enabler	Social opportunity
NoLo products make it more fashionable to say no to alcohol	Social influences	Enabler	Social opportunity
NoLo products are significantly lower in calories	Beliefs about consequences	Enabler	Reflective motivation
NoLo products are just another way for the alcohol industry to make money	Emotions	Barrier	Automatic motivation
To look after my mental/physical health	Intentions	Enabler	Reflective motivation
To avoid getting drunk	Reinforcement	Enabler	Automatic motivation
To avoid interactions with other drugs	Beliefs about consequences	Enabler	Reflective motivation
To help me consume fewer calories	Goals	Enabler	Reflective motivation
I am trying to drink less often	Goals	Enabler	Reflective motivation
To avoid doing something embarrassing when drunk	Emotions	Enabler	Automatic motivation
To stay safe when I am driving	Beliefs about consequences	Enabler	Reflective motivation
My friends/family like me to drink NoLo products	Social influences	Enabler	Social opportunity
To fit in better with others who are drinking	Social influences	Enabler	Social opportunity
It is fashionable to drink NoLo products	Social influences	Enabler	Social opportunity
I like the taste of NoLo products	Reinforcement	Enabler	Automatic motivation
Because I am pregnant/my partner is pregnant, or I want to become pregnant	Beliefs about consequences	Enabler	Reflective motivation
I drink alcohol for the effect, so these offer me nothing of interest	Reinforcement/emotion	Barrier	Automatic motivation
They are not widely available in my area	Environmental context/resources	Barrier	Physical opportunity
I have not heard of these products	Knowledge	Barrier	Psychological capability
I do not like the way they taste	Reinforcement	Barrier	Automatic motivation
Never occurred to me to try these products	Knowledge	Barrier	Psychological capability
I prefer to stick to water or soft drinks (e.g., soda, juice)	Reinforcement	Barrier	Automatic motivation
I wouldn't have a good time	Reinforcement/emotion	Barrier	Automatic motivation
They are too expensive for what they are	Environmental context/resources	Barrier	Physical opportunity
My friends/family prefer me to drink alcoholic products	Social influences	Barrier	Social opportunity

Abbreviations: COM‐B, capability, opportunity, motivation, behaviour; NoLo, no and low alcohol; TDF, Theoretical Domains Framework.
