# Supplementary material for: Barriers to the use of no and low alcohol products in high‐risk drinkers
Source: Drug Alcohol Rev. 2025 Feb 9;44(3):842–57. doi: 10.1111/dar.14006 (PMC11886542; doi:10.1111/dar.14006)
Supplement: Supplementary file 1 — FIGURE S1: Impact of age on ever use of no and low alcohol products compared to never use, controlling for other variables in model 1—Table 2. FIGURE S2: Impact of age on use of no and low alcohol products in the last 12 months compare to ever use of no and low alcohol products, controlling for other variables in model 1—Table 2. TABLE S1: NoLo use by respondent country. TABLE S2: Demographic factors across drinker categories relative to NoLo use. [file DAR-44-842-s001.docx]

Figure S1: Impact of age on ever use of no and low alcohol products compared to never use, controlling for other variables in model 1 – Table 2.


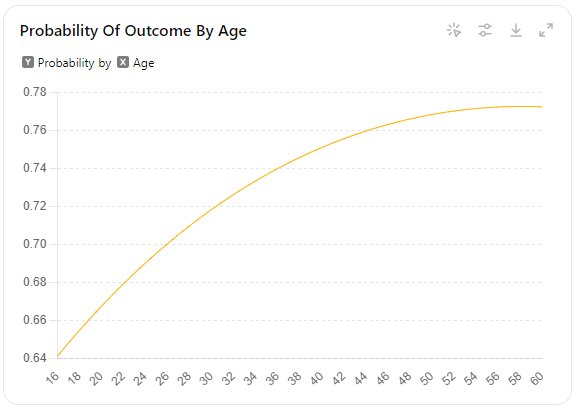


Figure S2: Impact of age on use of no and low alcohol products in the last 12 months compare to ever use of no and low alcohol products, controlling for other variables in model 1 – Table 2.


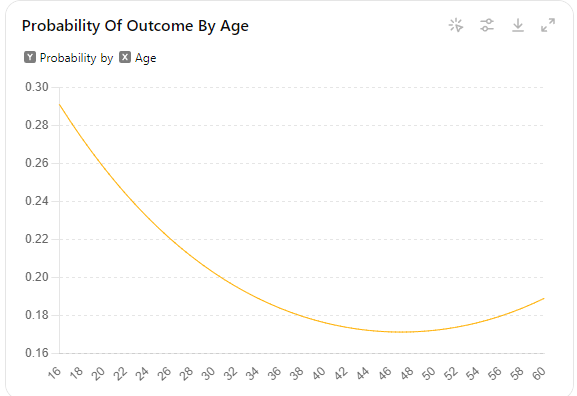


**Table S1:** *NoLo use by respondent country*

| Country | **Whole sample, N** | % | Never used NoLo, N | % | Used NoLo >12 months ago, N | % | Used NoLo <12 months ago, N | % |
| --- | --- | --- | --- | --- | --- | --- | --- | --- |
| **Total** | 30,033 | 100 | 9491 | 31.6 | 4855 | 16.2 | 15,687 | 52.2 |
| Australia | 1378 | 4.6 | 712 | 51.7 | 211 | 15.3 | 455 | 33.0 |
| Austria | 414 | 1.4 | 131 | 31.6 | 66 | 15.9 | 217 | 52.4 |
| Belgium | 162 | 0.5 | 61 | 37.7 | 31 | 19.1 | 70 | 43.2 |
| Brazil | 2702 | 9 | 1017 | 37.6 | 453 | 16.8 | 1232 | 45.6 |
| Canada | 239 | 0.8 | 119 | 49.8 | 60 | 25.1 | 60 | 25.1 |
| Colombia | 642 | 2.1 | 188 | 29.3 | 105 | 16.4 | 349 | 54.4 |
| Denmark | 274 | 0.9 | 77 | 28.1 | 51 | 18.6 | 146 | 53.3 |
| Finland | 727 | 2.4 | 241 | 33.1 | 111 | 15.3 | 375 | 51.6 |
| France | 282 | 0.9 | 131 | 46.5 | 38 | 13.5 | 113 | 40.1 |
| **Germany** | **12,183** | **40.6** | **2597** | **21.3** | **1747** | **14.3** | **7839** | **64.3** |
| Hungary | 687 | 2.3 | 216 | 31.4 | 117 | 17.0 | 354 | 51.5 |
| Ireland | 197 | 0.7 | 82 | 41.6 | 27 | 13.7 | 88 | 44.7 |
| Italy | 156 | 0.5 | 76 | 48.7 | 12 | 7.7 | 68 | 43.6 |
| Mexico | 1016 | 3.4 | 363 | 35.7 | 192 | 18.9 | 461 | 45.4 |
| **Netherlands** | **455** | **1.5** | **103** | **22.6** | **58** | **12.7** | **294** | **64.6** |
| New Zealand | 4682 | 15.6 | 1722 | 36.8 | 885 | 18.9 | 2075 | 44.3 |
| **Poland** | **370** | **1.2** | **44** | **11.9** | **35** | **9.5** | **291** | **78.6** |
| Spain | 149 | 0.5 | 41 | 27.5 | 24 | 16.1 | 84 | 56.4 |
| **Sweden** | **206** | **0.7** | **43** | **20.9** | **45** | **21.8** | **118** | **57.3** |
| **Switzerland** | **274** | **0.9** | **71** | **25.9** | **42** | **15.3** | **161** | **58.8** |
| United Kingdom | 1563 | 5.2 | 650 | 41.6 | 308 | 19.7 | 605 | 38.7 |
| United States | 1275 | 4.2 | 806 | 63.2 | 237 | 18.6 | 232 | 18.2 |

Note: Top five countries where respondents ticked yes to NoLo use in the last 12 months are highlighted in bold. NoLo, no and low alcohol.

**Table S2:** Demographic factors across drinker categories relative to NoLo use

| Drinker status |  | Non- drinker | | | Low risk | | | Increasing risk | | | Higher risk | | | Possible dependence | | |
| --- | --- | --- | --- | --- | --- | --- | --- | --- | --- | --- | --- | --- | --- | --- | --- | --- |
| Demographic | No-Lo use | Never used NoLo | Used NoLo >12 months ago | Used NoLo <12 months ago | Never used NoLo | Used NoLo >12 months ago | Used NoLo <12 months ago | Never used NoLo | Used NoLo >12 months ago | Used NoLo <12 months ago | Never used NoLo | Used NoLo >12 months ago | Used NoLo <12 months ago | Never used NoLo | Used NoLo >12 months ago | Used NoLo <12 months ago |
| Whole sample |  | 1470 | 880 | 1267 | 4545 | 2326 | 8157 | 2517 | 1177 | 4736 | 489 | 244 | 841 | 460 | 219 | 663 |
| Gender |  |  |  |  |  |  |  |  |  |  |  |  |  |  |  |  |
| Cis man | 17,857 (59.5) | 809 (55.0) | 464(52.7) | 698(55.1) | 2496(54.9) | 1278(54.9) | 4421(54.2) | 1673(66.5) | 802(68.1) | 3179(67.1) | 325(66.5) | 175(71.7) | 592(70.4) | 307(66.7) | 159(72.6) | 450 (67.9) |
| Cis woman | 11,206 (37.3) | 583(39.7) | 382(43.4) | 525(41.4) | 1861(40.9) | 974(41.9) | 3528(43.3) | 771(30.6) | 339(28.8) | 1432(30.2) | 143(29.2) | 59(24.2) | 228(27.1) | 130(28.3) | 53(24.4) | 189(28.5) |
| Trans/non binary/other | 970 (3.2) | 78(5.30) | 34(3.9) | 44(3.5) | 188(4.1) | 74(3.2) | 208(2.5) | 73(2.9) | 36(3.1) | 125(2.6) | 21(4.3) | 10(4.1) | 21(2.5) | 23(5.0) | 7(3.2) | 24(3.6) |
| Age, years |  |  |  |  |  |  |  |  |  |  |  |  |  |  |  |  |
| 16-25 | 7129 (23.7) | 304(20.7) | 118(13.4) | 196(15.5) | 1101(24.2) | 399(17.2) | 1636(20.1) | 894(35.5) | 287(24.4) | 1273(26.9) | 193(39.5) | 62(25.4) | 239(28.4) | 180(39.1) | 44(20.1) | 195(29.4) |
| 26-35 | 9221 (30.7) | 307(20.9) | 210(23.9) | 328(25.9) | 1317(29.0) | 647(27.8) | 2529(31.0) | 800(31.8) | 374(31.8) | 1804(38.1) | 128(26.2) | 70(28.7) | 284(33.8) | 127(27.6) | 55(25.1) | 231(34.8) |
| 36-45 | 6228 (20.7) | 323(22.0) | 206(23.4) | 297(23.4) | 951(20.9) | 537(23.1) | 1742(21.4) | 412(16.4) | 268(22.8) | 876(18.5) | 96(19.6) | 57(23.4) | 172(20.5) | 77(16.7) | 72(32.9) | 132(19.9) |
| 46+ | 7455 (24.8) | 536(36.5) | 346(39.3) | 446(35.2) | 1176(25.9) | 743(31.9) | 2250(27.6) | 411(16.3) | 248(21.1) | 783(16.5) | 72(14.7) | 55(22.5) | 146(17.4) | 76(16.5) | 48(21.9) | 105(15.8) |
| Ethnicity (N=29,745) |  |  |  |  |  |  |  |  |  |  |  |  |  |  |  |  |
| White | 25,561 (85.9) | 1163(80.6) | 707(80.9) | 1083(86.2) | 3738(83.2) | 1966(85.2) | 7081(87.4) | 2102(85.0) | 1002(86.0) | 4182(89.0) | 416(86.0) | 217(88.9) | 728(87.5) | 378(83.6) | 192(88.5) | 572(87.2) |
| Other ethnicity | 4184 (14.1) | 280(19.4) | 167(19.1) | 173(13.8) | 757(16.8) | 342(14.8) | 1024(12.6) | 371(15.0) | 163(14.0) | 517(11.0) | 68(14.0) | 27(11.1) | 105(12.6) | 74(16.4) | 25(11.5) | 84(12.8) |
| Employment status (N=30,017) |  |  |  |  |  |  |  |  |  |  |  |  |  |  |  |  |
| Full time | 16,744 (55.8) | 651(44.4) | 408 (46.4 | 687(54.3) | 2509(55.2) | 1294(55.6) | 4763(58.4) | 1442(57.3) | 657(55.8) | 2781(58.7) | 245(50.1) | 127(52.0) | 473(56.3) | 217(47.2) | 122(55.7) | 350(52.8) |
| Part-time | 5604 (18.7) | 249(17.0) | 147(16.7) | 238(18.8) | 767(16.9) | 458(19.7) | 1626(19.9) | 425(16.9) | 216(18.4) | 886(18.7) | 93(19.0) | 53(21.7) | 165(19.6) | 102(22.2) | 42(19.2) | 132(19.9) |
| Not-working | 7669 (25.5) | 566(38.6) | 325(36.9) | 341(26.9) | 1266(27.9) | 574(24.7) | 1765(21.6) | 649(25.8) | 304(25.8) | 1067(22.5) | 151(30.9) | 64(26.2) | 202(24.0) | 141(30.7) | 55(25.1) | 181(27.3) |

NoLo, no and low alcohol.
